# Closeness to Parents and Experiencing Threats with COVID-19 Mediates the Link between Personality and Stress among Adolescents

**DOI:** 10.3390/ijerph18126358

**Published:** 2021-06-11

**Authors:** Ewa Gurba, Alicja Senejko, Grzegorz Godawa, Alicja Kalus

**Affiliations:** 1 Department of Philosophy, The Pontifical University of John Paul II, 31-002 Cracow, Poland; ewa.gurba@upjp2.edu.pl; 2 Institute of Psychology, University of Wrocław, 50-137 Wrocław, Poland; alicja.senejko@uwr.edu.pl; 3 Department of Social Sciences, The Pontifical University of John Paul II, 31-002 Cracow, Poland; grzegorz.godawa@upjp2.edu.pl; 4 Institute of Psychology, University of Opole, 45-551 Opole, Poland

**Keywords:** adolescence, COVID-19, family relationship, personality, stress

## Abstract

Purpose: many researchers have already established that the 2019 COVID-19 pandemic poses a threat to adolescent psychological health. Studies on the COVID-19 pandemic mainly focus on individual psychological consequences, such as anxiety, depression or stress. The presented study added a family context to psychological analyses of the COVID-19 pandemic in adolescence. We examined the mediational effects of closeness to parents and perceived pandemic-related threats to relationships between personality (emotional stability and agreeableness) and stress in adolescents. Methods: in total, 413 students from secondary schools in southern Poland completed questionnaires measuring stress, personality, closeness to parents and experiencing threats with COVID-19. Results: the results demonstrated that closeness with parents in conjunction with experiencing family-related threats and threats related to lifestyle changes were mediators between adolescent personality traits and the intensity of the stress experienced. Conclusions: closeness with parents and threats experienced with COVID-19 mediate relationships between personality traits (emotional stability and agreeableness) and the intensification of stress in adolescents.

## 1. Introduction

The threat of COVID-19 infection has suddenly emerged in all societies. The World Health Organization recognized the coronavirus disease 2019 (COVID-19) as a public health emergency of international concern [1]. The relatively poor knowledge of the virus, the ease of infection, the speed with which it spreads and the threat to life made this situation a source of stress and even trauma for people of different ages [2,3].

The first case of COVID-19 infection in Poland was confirmed on 4 March 2020 and, on March 13, a state of epidemic emergency was declared. Mass events were banned, and cultural and other activities involving larger social groups have been limited [4]. One of many early preventive measures taken against the spread of the virus was the government’s decision to close all schools (11 March 2020). Subsequently, starting from March 2020, remote education was implemented and developed throughout 2020 and 2021 [5]. Psychological burdens, social isolation and loneliness have been among the many problems emphasized by Polish adolescents (aged 11–18), which have been also experienced due to online education [5].

Researchers emphasize that people who are at a persistent risk of infectious diseases and who face a degree of uncertainty tend to suffer from higher rates of depression, anxiety, panic attacks or other mental health problems, including suicide [6,7]. Many researchers assume that the COVID-19 pandemic poses a serious challenge to people’s mental health [6]. There is therefore an urgent need for research into the consequences of a pandemic in the area of mental health in societies and to search for individual and relational resources to cope with these problems.

Adolescence is a period in which specific biological changes are related to the development of the brain and body [8]. Along with maturation, which might be a source of frequent and highly stressful experiences in young people, a significant increase in the reactivity of the hypothalamic–pituitary–adrenal (HPA) axis occurs [9,10]. Research results indicate that exposure to various stressors leads to a stronger or more prolonged response of stress hormones in adolescents, compared with adults [11,12,13,14]. It is a time of intensive peer relationships, usually providing opportunities to gain support and relieve stress and tension in fun activities. It was found that, in order to suppress the spread of COVID-19 and, consequently, reduce the number of deaths, it was necessary to limit social contact as much as possible, which, for teenagers, meant they could only meet their peers on social media [15].

In the pandemic situation, young people have been forced to limit their social relations and to only contact their peers online, while at the same time being forced to be in regular contact with their close family members. Although parents continue to be an important source of support for adolescents, any situations of coercion are perceived by them as restrictions on their pursuit of autonomy [16,17,18]. The current difficulty in meeting the need for autonomy and the increasing helplessness may also contribute to their verification of the quality of relations with their parents—the mother and the father—and the degree of closeness to each of them. The quarantine has also forced the adults to work remotely. In a situation of weakened or conflicting relationships between adolescents and parents, a greater intensity of contact may increase the severity of domestic problems [19]. Additionally, uncertainty about their near and distant future can also be stressful for teenagers and, yet, formulating goals and building a vision of one’s life provides the basis for the formation of identity, which is a fundamental developmental task for adolescents [20]. Not only the fear of themselves or their relatives becoming ill, but also the way of life imposed on them, depriving them of the basic needs appropriate for their developmental period, could be additional source of stress related to the pandemic. On a daily basis, these needs are usually satisfied through specific patterns of behaviour, rituals and activities that constitute an adolescent’s style of functioning.

The extent to which the current pandemic situation is perceived as stressful may also be related to an adolescent’s personality traits. Therefore, the work presented here examines the links between adolescent personality traits and the closeness to parents—to the mother and father, the type of COVID-19 risks they experience and the level of stress they experience. As suggested by Cohen et al. [21], we define stress as a process in which environmental demands strain an organism’s adaptive capacity, resulting in both psychological and biological changes that could place a person at risk of illness. The presented research fills the gap in the study of correlations between teenagers’ personality traits and the stress they experience under pandemic conditions, taking into account social determinants such as their relationships with their parents.

In the area of developmental tasks, the arduous path of searching for one’s own identity is a stress-inducing factor for adolescents [22]. Changes in cognitive competences in the form of acquiring the ability to engage in logical and abstract thinking is an important tool for building one’s own identity; at the same time, however, people are encouraged to criticize themselves and others, which often becomes another source of stress [23]. Young people are now required to be able to plan their academic and professional future at a very early age. Another potential source of stress in this developmental period is the teenager’s normative opposition to the authority of their parents in favor of the authority of the peer group, especially in the context of the first intimate relationships [24,25]. The above-mentioned circumstances accompanying adolescence, along with the increased biological susceptibility of adolescents to stress, make adolescence a time of increased tensions and, often, perceived threats [26,27,28].

Adolescents’ personality traits may be relevant to the stress they experience in their interpersonal relationships. The types of stressors or the influence of additional variables (e.g., the quality of relationships with important people or the possibility of obtaining social support) might modify the relationships between personality traits and the level of stress [29,30].

The above-described psychological and social factors, together with the increased biological susceptibility to stress, makes adolescence a time of increased tensions and frequently perceived threats and, thus, increased susceptibility to the adverse consequences of the pandemic. Referring to Lazarus and Folkman’s [31] transactional model of stress, we assume that the level of stress associated with COVID-19 in adolescents is the result of the assessment of the type and the degree of the threats associated with the pandemic. There is still a demand for studies on the importance of the quality of interpersonal relationships in coping with stress [32], especially related to personality traits. Therefore, our study included both personal and social determinants of experiencing COVID-19-related stress. We focused on the importance of personality traits and closeness to their mother and father, as well as the types of risks and threats associated with COVID-19 experienced by adolescents that lead to the intensification of their stress.

### 1.1. Adolescents’ Closeness to Their Parents as a Predictor of Coping with Stress in the Times of the COVID-19 Pandemic

Close relationships, commonly characterized by a degree of intimacy, self-disclosure and coherence, are the most important sources of support for an individual in the process of regulating emotions [33]. Therefore, the quality of close relationships plays an important protective role, providing social resources for coping with difficult situations and reducing the intensity of stress [34]. This is because social ties encourage the development of resistance and more adaptive defence mechanisms, as indicated by the stress-buffering hypothesis [35]. In the context of our research on adolescents’ experience of COVID-19-related stress, we focus primarily on the closeness of adolescents to their parents as an important source of support in the context of the necessary isolation, when direct contacts with peers have been limited to online relations [16]. Research suggests that the negative impact of life stress on adolescents can be modified by warm, supportive and caring parents that can communicate well with their teenagers [36]. The protective effect of close relations between adolescents and their parents takes place despite the fact that the nature of teenagers’ relations with their parents is changing. Relationships based on authority and dependency are transformed into relationships based on respect and partnership; however, for important life issues, adolescents still refer to the authority of their parents [37,38], and close relationships with one or both parents provide an important source of support to them. The results of longitudinal studies on adolescents show the importance of close relationships with parents, primarily with mothers, for stress relief [16,39], including the case of long-term effects in the form of adolescent depression [16]. Mothers have been perceived by adolescents as more supportive and warm than fathers [38,40]. However, adolescent personality traits such as extraversion [41], agreeableness [42] and emotional stability [43] predict close, warm relationships based on parental support.

### 1.2. The Present Study—Personality, Closeness with Parents and COVID-19 Stress in Adolescents

The aim of our research is to determine the effects of selected personality traits, closeness to parents and types of pandemic-related threats on the intensity of the stress experienced by adolescents in connection with COVID-19. The specific nature of the stressful situation considered was that the risk of contracting the coronavirus only affected a specific respondent and their family members but resulted in the maximum limitation of direct contact with other people outside the home. Among the types of threats connected with COVID-19, and indicated by the adolescents examined, we distinguished the following: (1) threats to life (TTL), which included the threat of the adolescent’s own illness, death and illness of family members, as well as the deterioration of the family’s financial situation; (2) family threats (FT), which included excessive control by parents and conflicts with parents and siblings; (3) threats to lifestyle (TTLS), which included situations of the restriction of freedom of movement, contact with peers and use of various services. Since the typical demands of young people for direct contact with their peers, freedom of movement in the fulfilment of everyday tasks and closeness and contact with their beloved ones have been significantly limited in the pandemic situation, we predicted that threats in this area (TTLS) would play an important role in the intensification of pandemic stress in adolescents [44]. The frustration of the above-mentioned important needs of adolescents may, in turn, contribute to family conflicts (FT), thus increasing the pandemic stress. TTL was not expected to have much significance for the intensification of stress experienced by young people, due to the above-mentioned normative developmental changes.

Due to the specificity of the above-described pandemic-related threats, we focused on two personality dimensions: emotional stability and agreeableness and their relationship to experienced levels of stress. Emotional stability is the opposite pole of neuroticism, which means susceptibility to experiencing various negative emotions such as fear, anger, dissatisfaction and a sense of guilt; therefore, it promotes the experience of severe stress and is an obstacle to dealing effectively with difficult situations [45]. The opposite is true for people with low neuroticism, i.e., those who are emotionally stable. They exhibit the ability to control their emotions, which makes them resistant to difficulties and stress [46,47]. Therefore, we predict that emotionally stable teenagers will experience less stress. By protecting against potential social conflicts, emotional stability can foster closeness in adolescents’ relationships with their mothers and fathers.

For adolescents, especially more social adolescents, the situation of forced quarantine and the inability to contact their peers directly can be difficult. Since agreeableness is linked to the need for close interpersonal contact and positive social relations, it can play both a positive role in coping, mainly by helping others in the pandemic situation, and a negative role in circumstances of enforced restrictions on social contacts [28,48]. Close relationships provide support and provide the anticipation of receiving support when it is needed, thereby protecting against an increase in stress and possibly contributing to making young people less at risk from a pandemic [28]. We predicted that emotionally stable teenagers would experience less stress, partly due to close relations with their mothers and fathers. We also expected that closeness with parents and perceived pandemic threats would serve as mediators in the relationship between emotional stability and agreeableness, and the level of experienced stress.

## 2. Method

### 2.1. Participants

The participants comprised 412 young people aged between 14 and 19 years, with a mean age of 17 years, of whom 59% were women and 41% were men. The participants were pupils of five secondary schools located in Krakow, of whom 59% lived in a large city and 41% in a small town or village. Among all the respondents, 81% resided with both parents, 11% only with the mother and the remaining 8% resided with only the father, the grandparents or only with their siblings, or alternately with the mother and the father.

Most of the parents of the study participants had higher education (mothers: 70%, fathers: 59%), 22% of the mothers and 21% of the fathers had secondary education and 8% of the mothers and 20% of the fathers had vocational education.

All students from five schools in Kraków were invited to participate in the study. All the pupils were of Polish origin. No participant was excluded of those who completed all the tests.

### 2.2. Measures

The study used four questionnaires and a metric, allowing us to collect basic demographic data from the respondents. The following methods were used:

*Personality*. The short IPIP-BFM-20 questionnaire, which was an abridged Polish version of the IPIP-BFM-50 by Goldberg [30], was used to measure the Big Five personality traits. The questionnaire consisted of 20 items for five scales (extraversion, agreeableness, conscientiousness, emotional stability and intellect), with a 5-point response scale from 1, “a completely inaccurate description of me”, to 5, “a completely accurate description of me”. The Polish version was prepared by Topolewska, Skimina, Strus, Cieciuch and Rowiński [49]. Example items included the following: “I keep in the background” (extraversion), “I sympathize with others’ feelings” (agreeableness), “I get chores done right away” (conscientiousness), “I worry about things” (emotional stability) and “I have a rich vocabulary” (intellect).

The Scale of Closeness to Biological Parents by Regnerus [50] consists of six items separately describing interactions with the mother and father. The Polish version was prepared by E. Gurba. Respondents were asked to evaluate their current relationship with their mother and father by reporting the frequency of six parent-child interactions. For each parent figure, these six items were coded and summed into a parental closeness index. Example items included the following: “How often do you talk openly with your parent about things that are important to you?” and “Would your parent help you if you had a problem?” The response categories ranged from never (1) to always (5).

*Intensification of stress*. The Perceived Stress Scale (PSS) 10, by Cohen, Kamack and Mermelstein [51], was adapted by Juczyński and Ogińska-Bulik [52]. The PSS-10 measures the global stress in a given life situation as well as the difficulty in coping with it, alongside the intensity of negative emotions over the last month. It consists of 10 statements assessed on a 5-point Likert scale (from never to very often). Example items included the following: “In the last month, how often have you been upset because of something that happened unexpectedly?” and “In the last month, how often have you been able to control irritations in your life?” The level of stress measured in our study was denoted as STR.

*Experiencing threats with COVID-19.* The Pandemic Threat Assessment Questionnaire (PTAQ) by Gurba, Godawa and Senejko consists of 18 items describing pandemic-related situations that could be perceived as potentially threatening in the opinion of adolescents. The respondent assessed these situations on a five-point scale (1—not threatening at all; 2—slightly threatening; 3—moderately threatening; 4—significantly threatening; 5—very strongly threatening), indicating the extent to which each of the described situations related to the COVID-19 pandemic would be burdensome and threatening. The metric contained questions about gender, age, family of origin and place of residence.

Using the exploratory factor analysis method of Principal Components Analysis (PCA) for 15 items, three threatening factors were distinguished: (1) threats to life (TTL), including the feeling that the health and life of loved ones is in danger and the loss or threat of losing a job for their parents, etc. (examples of items: risk of coronavirus infection; threats to loved ones’ health and lives); (2) family threats (FT), referring to frustration due to crowds in the place of residence, conflicts with parents, siblings, etc. (examples of items: intensification of conflicts between parents; increased control by parents due to their constant presence); (3) threats to lifestyle (TTLS), referring to the inability to meet vital life needs related to direct contacts with peers, free movement and the implementation of routine and life-stabilizing behaviors (examples of items: not being allowed to use various services; being forced to change one’s plans in life). Each of the factors were explored with five questionnaire items (Table 1).

The reliability of the PTAQ-1 was satisfactory (FT Cronbach’s α = 0.79; TTL Cronbach’s α = 0.80; TTLS Cronbach’s α = 0.78). The other values were as follows: χ2 (19) = 187.07, *p* < 0.001, SRMR = 0.04, RMSEA = 0.086, CFI = 0.94. Factor loadings ranged from 0.32 to 0.78.

### 2.3. Procedure

The study was conducted during the pandemic in the third and fourth week after the closure of schools (April 2020) when lessons were streamed online and the whole of Polish society was subjected to a mandatory quarantine (children and young people did not attend schools, and adults worked at home wherever possible). Places of residence could only be left when going to places of work, if necessary, to go shopping for groceries or to go for a walk with an animal. The sheet with the questionnaires to be completed was placed on the websites of five secondary schools in Krakow with the consent of the school management. It was accessible to students for a fortnight. Admittedly, we sent a request to participate in the survey to the management of all secondary schools in Krakow, giving a two-week time to decide on the participation of pupils from these schools in the survey. Such a short deadline was due to the fact that we wanted to start the research as early as possible, at the beginning of the Lockdown. It is likely that shortened time to decide on participation in the survey, which we proposed to the school management, resulted in the fact that finally only five secondary schools signed up for the participation of their students in the survey. Parents gave their consent for the young people to complete the survey. The survey was voluntary and anonymous. The respondents were informed that they could stop completing questionnaires at any time. It took approximately 30 min to complete the survey. Questions included in the questionnaires did not affect the well-being of the respondents. Respondents were assured of complete anonymity. The survey was approved by the Ethics Committee of one of the Polish universities.

## 3. Results

Arithmetic means and standard deviations were calculated for the values of the individual variables, as presented in Table 2.

The following data analyses were carried out: simple correlations between the individual variables, the Structural Equation Modeling (SEM) method for the effects of emotional stability (STA) and agreeableness (AGR) on the severity of stress (STR), with the intermediating effect of family threats (FT) and (TTLS) concerning lifestyle and closeness to the father (CTF) and mother (CTM) (standardized coefficients).

Prior to the analysis, cases with deficiencies were removed, which reduced the number of respondents from N = 412 to N = 405, as there was no indication of the type of data input (the number of cases removed was small). The reliability analysis using Cronbach’s alpha method showed the satisfactory reliability and consistency of the variables created. Although all variables showed deviations from the normal distribution, the critical statistics of the multidimensional normal distribution did not exceed 5; therefore, it was decided to use the parametric method of the highest reliability (ML), and the estimation of all coefficients was based on that method. Table 2 contains the descriptive statistics of the analyzed variables.

For the complete picture of the obtained data, we calculated the simple correlations between the individual variables (Table 3). In turn, the calculations of standardized and non-standardized path coefficients with their relevance: detailed direct and indirect effects (Table 4), are already an introduction to the Structural Equation Modeling (SEM) method.

In order to assess whether the theoretical model is consistent with the data obtained from the survey, an analysis was carried out using the Structural Equation Modelling (SEM) method. The analysis aimed to identify the extent to which adolescents’ personality traits of emotional stability and agreeableness allowed the prediction of the severity of pandemic-related stress; the importance of closeness to the father and mother and the types of threats (threats to family (FT) and lifestyle changes (TTLS)) were taken into account as mediating variables in this relationship.

Adolescents’ closeness to their mother (CTM) both directly and indirectly (intermediated by family threats (FT)) had an effect on the intensification of stress (STR). Closeness to the mother (CTM) directly reduced the level of family threats (FT) and also promoted closeness to the father (CTF), reducing the level of family threats. In the analyzed model, however, the variable of closeness to the mother explained only a small percentage of the variance.

**Figure 1:**
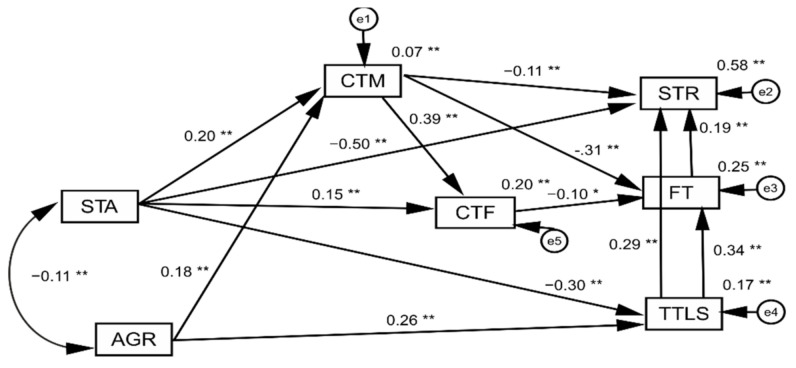
Result of structural modelling for the effects of emotional stability (STA) and agreeableness (AGR) on the severity of stress (ST)R, with the intermediating effect of family threats (FT and TTLS) concerning lifestyle and closeness to the father (CTF) and mother (CTM) (standardized coefficients). * *p* < 0.05, ** *p* < 0.01.

Closeness to the father only had an indirect effect on the intensification of stress (STR) by lowering the level of family threats. The effect of closeness to the father, compared with closeness to the mother in relation to family threats, was much weaker. Threats to lifestyle (TTLS), related to lifestyle and family threats (FT) intensified stress (STR) in the pandemic situation. Threats to lifestyle intensified the experience of family threats (FT) and, through their intermediation and also directly, intensified the stress associated with COVID-19. Family threats were also relevant to the intensification of stress (Figure 1).

## 4. Discussion

The study examined the links between adolescents’ personality traits, their closeness to the mother and father, the types of threats related to COVID-19 and the intensity of the stress they experienced. Taking into account the available literature describing developmental patterns in adolescence, the specificity of teenagers’ relationships with their parents and peers, the characteristics of personality traits and the importance of these factors in adolescents’ experience of highly stressful situations, a theoretical model was constructed [24,46]. It was expected that, of the five personality traits assessed in the study, two factors—emotional stability and agreeableness—would play a significant role. The assumed predictions were confirmed. Emotional stability was found to be a direct positive predictor of closeness to the mother and father, but it also lowered the level of threats in the category threats to lifestyle, and significantly and directly reduced the intensity of the stress experienced. Emotional stability also lowered the level of family threats, which was a predictor of the intensity of stress, by increasing closeness to the father and mother. In the five-factor model of personality that was adopted, emotional stability was the opposite pole of the dimension of neuroticism. Emotionally stable individuals are calm, relaxed, able to control their emotions and do not easily fall into negative emotional states and therefore are resistant to difficulties and stress [47]; the obtained results were fully consistent with the above characteristics of emotional stability. Agreeableness exerted a less significant effect in the model than emotional stability. We found that agreeableness was conducive to closeness to the mother, as also shown by other studies [42,53]. At the same time, agreeableness in our study increased the level of threats to lifestyle and thereby contributed to an increase in the level of stress experienced by adolescents. Agreeableness is a personality dimension that describes attitudes towards other people, the need for contact with them and positive social relations. People with a high intensity of agreeableness are empathic, highly sensitive to others’ concerns and willing to help them. This trait promotes teenagers’ closeness not only to their parents but above all to their peers [37,41]. Other studies have shown that a high level of agreeableness has been correlated with a high level of both perceived and received social support [11]. Meanwhile, the pandemic situation and forced social isolation and quarantine have made it much more difficult to both provide and receive social support. The essence of the threat to lifestyle that we investigated is the restrictions affecting adolescents and experienced mainly with regard to relationships which form part of the ritual of everyday life. In a non-pandemic situation, social relations, mainly with peers, perform a variety of functions such as substituting the family, stabilizing personality, shaping one’s own self-esteem, developing social competences or adopting role models [54]. It is therefore not surprising that agreeableness intensified the threats described above and, through them, had an effect on intensifying stress in our study. A similar correlation was noted when the threat was related to social conflicts [35]. The results obtained confirm the suggestions made by authors who highlight the importance of the specific nature of stressful situations for predicting human behaviour [55] and may explain the inconsistency of the results of studies on the correlation between individual personality traits and the intensity of the stress being experienced [48,53,56,57].

Using the Pandemic Threat Assessment Questionnaire (PTAQ), we estimated three categories of pandemic threats identified by means of factor analysis: (1) threats to life (TTL), consisting, among other things, of the threat of illness, death and illness of family members, as well as the deterioration of the family’s financial situation; (2) family threats (FT), consisting of excessive control by parents and conflicts with parents and siblings; (3) threats to lifestyle (TTLS), consisting of situations of reduced contact with peers, freedom of movement, the use of various services and disruption of previous activities. We found that, in the context of theoretical and empirical analyses, two categories of threats—FT and TTLS—intensified the stress experienced by Polish adolescents in the epidemic situation. As predicted, threats to life did not have a significant effect on the intensity of epidemic stress. This may indicate an underestimation by adolescents of the dangers directly related to their health and life [58].

The pandemic situation is a major challenge, mainly for people’s social functioning. In the case of adolescents, the limitations and restrictions related to COVID-19 may pose a threat to the course of their developmental processes during adolescence. Thus, despite a relatively small group of respondents representing different stages of adolescence, which stemmed from the limited two-week access to the questionnaires to control for the similarity of pandemic conditions, this study presents a number of insights. As the results of our research show, the disruption of the daily functioning pattern is experienced by adolescents as a threat to lifestyle, which intensifies the experience of family threats. Relationships with parents and peers are particularly sensitive areas of adolescents’ functioning. The frustration of adolescents’ basic needs for autonomy and social relationships, mainly with their peers, as the result of forced quarantine, may therefore result in increased family problems, such as conflicts with parents and siblings, excessive parental control, lack of respect for privacy, etc., especially when parents are perceived by adolescents as not providing them with support [59,60,61]. Both categories of threats added to the intensification of stress experienced in the pandemic situation, which is consistent with Lazarus and Folkman’s transactional model of stress [31].

The results of our research confirm the correlations adopted in our model between adolescents’ closeness to the mother and father and the intensity of stress related to COVID-19. According to other studies, close relationships are an important source of support for people experiencing difficult situations [62]. As it turned out, close relationships with parents to some extent protected adolescents from experiencing the severe stress associated with the pandemic. Additionally, the results of other studies presented in the literature suggest that the negative impact of life stress on adolescents can be modified by warm, supportive and caring parents that can communicate well with their teenagers [36,63,64]. The protective effect of close relations between adolescents and their parents takes place despite the fact that the nature of teenagers’ relations with their parents changes from relationships based on authority and dependency to relationships of partnership [38]. In the analyzed model, closeness to the mother was found to possibly prevent adolescents from experiencing severe stress in dangerous situations along three paths. Closeness to the mother provides an adolescent with (1) emotional support, which is the source of their resilience and thus protects them from strong stress experiences (this mechanism is included in the stress-buffering hypothesis [17]); (2) the sense of security, which is experienced through closeness to the mother, contributes to the fact that difficult situations are not perceived by the adolescent as highly threatening. In the pandemic quarantine situation, the constant presence of the parents, even misunderstandings with them or their siblings, and the sense of being subject to parental control, constituted a significant nuisance to adolescents in close relationships with their parents, especially the mother. However, the security experienced in the relationship with parents protected adolescents from seeing these situations as a threat to the functioning of the family system or to themselves; (3) closeness to the mother can also provide a basis for building a sense of closeness to the father, which in turn can help a teenager to deal constructively with difficult situations [38].

## 5. Conclusions

Summarizing the results, it can be stated that among the variables included in the theoretic model, the personality trait that played the most significant role in explaining the intensity of stress experienced by adolescents in the initial acute phase of the COVID-19 pandemic (when there was a restrictive quarantine in Poland) was emotional stability. Its level, in combination with closeness to parents, directly reduced the intensity of stress experienced by adolescents. The second of the analyzed traits—agreeableness—on the one hand favored adolescents’ closeness to their mother and could thus reduce the intensity of the stress experienced, however, on the other hand, could cause the changes in the lifestyle enforced by the pandemic to be experienced by young people as more threatening. The results of our research have shown that closeness to parents can be an important factor, reducing both the strength of stress and potential threats related to the functioning of the family in difficult life circumstances, such as a pandemic [2]. Therefore, as part of the preventive strategies aimed at children and adolescents with goals of their protection and optimal functioning in the face of threats, closeness with parents should be taken into account. Another important result is that the factors taken into account in the research, such as closeness with parents, personality traits, types of emotional reactivity and agreeableness, were not related to experiencing threats from the LT life threatening category by teenagers and adolescents, nor were they related to the physical and material violation of the safety of the respondents and their relatives. This can be an important signal for social institutions, including the family, regarding which direction educational goals and care for minors should be directed, because the results of our research may indicate an underestimation by children and adolescents (especially sensitive and conciliatory young people) of the dangers directly related to their health and life, resulting from functioning in the conditions of a pandemic [65]. The prediction of distress tolerance was based on the feeling of loneliness and self-handicapping in students.

The very important result of our research is that it shows the extremely important role of the TTLS lifestyle threats—i.e., blocking or severely hindering the possibility of implementing behaviors through which young people typically satisfied their needs, passions and life values—in influencing the functioning of teenagers and adolescents in a pandemic. Despite the fact that the closeness with parents did not influence this kind of threat, these threats intensified the stress and the possibility of the emergence of family threats. Our results can therefore be used by both parents and social and even governmental institutions when preparing, for example, a plan to end the lockdowns, in order to first enable children and adolescents to form the behavior, mainly in the context of social relations, through which they realized their needs and passions before the pandemic. [41,55,65].

The obtained results emphasize the protective importance of closeness in family relations, especially between children and parents. The condition for this strengthening is building closeness based on the strength of family ties while maintaining the autonomy of individual family members. That means that educational activities supporting the family are necessary, with the aim of making families experiencing subsequent stages of the pandemic aware of the potential of a social group based on closeness.

These activities also protect against inappropriate educational attitudes from parents who reduce closeness to spending time together or equate closeness with unification. Efforts are also needed to formulate a new social model of closeness that could emerge from the pandemic.

The results of our research have shown that closeness to parents can be an important factor in reducing both the strength of stress and potential threats related to the functioning of the family in difficult life circumstances, such as a pandemic. Therefore, as part of the preventive strategies aimed at children and adolescents with the goal of their protection and optimal functioning in the face of threats, closeness with parents should be taken into account. Another important result is that the factors taken into account in the research, such as closeness to parents, personality traits, types of emotional reactivity and agreeableness, had an impact on the experience by young people of threats from the LT life hazards category, related to disturbing the physical and material security of the respondents’ existence and their loved ones. This can be an important signal for social institutions, including the family, in which educational goals and care for minors should be directed, because the results of our research may indicate an underestimation by children and adolescents (especially the sensitive and conciliatory) of the dangers directly related to their health and life, as a result of functioning in the conditions of a pandemic. The research carried out and presented here can help in this work, providing a good basis for further discussions.

### Limitations of the Study

Reflecting on the study allows us to see some of its weaknesses. First of all, attention should be drawn to the relatively small group of respondents. The specific conditions of remote learning, in which adolescents spend a large amount of time in front of the computer screen while participating in school lessons, discouraged them from participating in the research, which was also conducted online.

In addition, the respondents represented different stages of adolescence (14–19 years of age); however, due to the fact that the group of respondents was not large enough, it was not possible to carry out analyses taking into account developmental stages.

Another weakness of our research is the failure to take into account factors that may explain the obtained results, e.g., the characteristics of family dynamics before the pandemic, the socio-economic and cultural attributes of teenagers’ families, educational difficulties of the adolescents, the level of social acceptance, conditions during isolation or access to social support.

## Figures and Tables

**Table 1:** Factor structure of the Pandemic Threat Assessment Questionnaire (PTAQ). Rotated factorial loads (Varimax rotation).

	Item	1	2	3
KZ11	Increased number of conflicts with parents	0.82		
KZ17	Intensification of conflicts between parents	0.73		
KZ7	Increased control by parents due to their constant presence	0.71		
KZ8	Increased frequency of conflicts with siblings	0.69		
KZ4	Congestion of people in the place of residence	0.60		
KZ14	Sense of threat to own life		0.79	
KZ3	Risk of coronavirus infection		0.75	
KZ15	Anticipation of deterioration of the financial situation		0.70	
KZ10	Threats to loved ones’ health and lives		0.70	
KZ5	Loss or threat of loss of job by parents		0.67	
KZ1	Limited or broken direct contacts with peers			0.85
KZ13	Sense of loneliness in isolation			0.72
KZ9	Not being allowed to move freely			0.71
KZ2	Not being allowed to use various services			0.63
KZ6	Being forced to change one’s plans in life			0.52
	Own values	4.65	2.07	1.70
	% of variance	31.02	13.79	11.32
	A	0.79	0.80	0.78

**Table 2:** Descriptive statistics of variables (N = 405).

Acronym	Name	R	M	SD	Sk	Kurt	D	A
FT	Family Threats	0.00–5	2.19	1.00	0.57	−0.47	0.12 **	0.79
TTL	Threats to Life	0.00–5	2.95	0.99	0.09	−0.57	0.07 **	0.80
TTLS	Threats to Lifestyle	0.00–5	3.26	0.97	−0.44	−0.39	0.10 **	0.78
EXT	Extraversion,	1.00–5	3.07	1.06	−0.12	−0.86	0.09 **	0.84
INT	Intellect	1.25–5	3.65	0.71	−0.31	−0.30	0.10 **	0.66
STA	Emotional stability	1.00–5	2.53	0.97	0.30	−0.59	0.09 **	0.80
CON	Conscientiousness	1.00–5	3.04	0.88	−0.11	−0.48	0.07 **	0.72
AGR	Agreeableness	1.25–5	3.91	0.74	−0.83	0.75	0.13 **	0.70
CTM	Closeness to the mother	1.00–5	3.76	0.89	−0.91	0.50	0.13 **	0.80
CTF	Closeness to the father	1.00–5	3.23	1.05	−0.59	−0.55	0.12 **	0.85
STR	The level of stress	1.00–5	3.03	0.79	−0.11	−0.46	0.05 *	0.89

* *p* < 0.05, ** *p* < 0.01.

**Table 3:** Simple correlations between the individual variables (N = 405).

		1	2	3	4	5	6	7
1	FT							
2	TTLS	0.37 **						
3	STR	0.46 **	0.52 **					
4	CTM	−0.33 **	−0.03	−0.22 **				
5	CTF	−0.24 **	−0.07	−0.21 **	0.45 **			
6	STA	−0.25 **	−0.31 **	−0.64 **	0.14 **	0.20 **		
7	AGR	0.01	0.26 **	0.11 *	0.17 **	0.16 **	−0.10	

* *p* < 0.05, ** *p* < 0.01.

**Table 4:** Standardized and non-standardized path coefficients with their relevance: detailed direct and indirect effects.

Path	B	β	*p*
CTM<---STA	0.18	0.20	<0.001
CTM<---AGR	0.22	0.18	<0.001
CTF<---STA	0.16	0.15	0.001
TTLS<---STA	−0.30	−0.30	<0.001
TTLS<---AGR	0.34	0.26	<0.001
CTF<---CTM	0.47	0.40	<0.001
FT<---CTM	−0.35	−0.31	<0.001
FT<---CTF	−0.09	−0.10	0.037
FT<---TTLS	0.35	0.34	<0.001
STR<---CTM	−0.10	−0.11	0.002
STR<---STA	−0.41	−0.50	<0.001
STR<---TTLS	0.24	0.29	<0.001
STR<---FT	0.15	0.19	<0.001
STA<-->AGR	−0.08	−0.11	0.029
CTF<<---AGR	0.10	0.07	0.001
CTF<<---STA	0.09	0.08	0.001
FT<<---AGR	0.03	0.02	0.385
FT<<---STA	−0.19	−0.18	0.001
FT<<---CTM	−0.04	−0.04	0.035
STR<<---AGR	0.07	0.06	0.010
STR<<---STA	−0.12	−0.14	0.001
STR<<---CTM	−0.06	−0.07	0.001
STR<<---CTF	−0.01	−0.02	0.032
STR<<---TTLS	0.05	0.06	0.001

Note: statistical significance of indirect effects was calculated using bootstrap modeling for a 95% confidence interval; <--- direct effect; <<--- indirect effect.

## Data Availability

Not applicable.
